# Risk factors of major complications after flap surgery in the treatment of stage III and IV pressure injury in people with spinal cord injury/disorder: a retrospective cohort study

**DOI:** 10.1038/s41393-023-00944-9

**Published:** 2023-12-20

**Authors:** Carina Fähndrich, Armin Gemperli, Michael Baumberger, Michael Harder, Bianca Roth, Dirk J. Schaefer, Reto Wettstein, Anke Scheel-Sailer

**Affiliations:** 1 Swiss Paraplegic Research, Nottwil, Switzerland; 2 Faculty of Health Sciences and Medicine, University of Lucerne, Lucerne, Switzerland; 3 Center of Primary and Community Care, University of Lucerne, Lucerne, Switzerland; 4 Swiss Paraplegic Centre, Nottwil, Switzerland; 5 Department of Infectious diseases, Cantonal Hospital Lucerne, Lucerne, Switzerland; 6 Department of Plastic, Reconstructive, Aesthetic and Hand Surgery, University Hospital of Basel, Basel, Switzerland

**Keywords:** Risk factors, Health care

## Abstract

**Study design:**

Retrospective cohort study.

**Objectives:**

To identify risk factors associated with major complications after flap surgery in people with spinal cord injury or disorder (SCI/D) and stage III and IV pressure injury (PI).

**Setting:**

Swiss hospital specialized in the treatment of people with SCI/D using the Basel Decubitus Approach.

**Methods:**

We examined 60 risk factors for major postoperative complications in PIs over sacrum/coccyx, ischium or trochanter between 01/2016 and 12/2021. We performed descriptive analysis and computed global p-values using likelihood ratio tests adjusted for clustering of PIs in individuals.

**Results:**

We included 220 PI treatment procedure from 149 individuals. The study population consisted of 163 (74%) men, 133 (60%) traumatic SCI, 136 (58%) stage IV PI, 198 (90%) individuals with paraplegia, 93 (42%) with osteomyelitis, and 85 (39%) with recurrent PI. Major complications 42 (19%) occurred more often in individuals with stage IV PI (*p* < 0.01), individuals without osteomyelitis (*p* < 0.03), and individuals with pathological blood concentrations of cystatin c (*p* < 0.028), calcium (*p* < 0.048), and vitamin B12 (*p* < 0.0049) as well as normal blood concentrations of HbA1c (*p* < 0.033). Immobilization (*p* < 0.0089) and hospital stay (*p* < 0.0001) of individuals with major complications was longer.

**Conclusion:**

In the Basel Decubitus Approach, stage IV PI, absence of osteomyelitis, reduced vitamin B12 and calcium, elevated cystatin c, and normal HbA1c should be addressed to reduce major complications.

## Introduction

Pressure injury (PI) is the second most common condition associated with spinal cord injury or spinal cord disorder (SCI/D) [1]. It is a potentially life-threatening condition that significantly limits quality of life [1–3]. PIs generate the highest cost among all SCI/D complications [4]. Reported prevalence rates of PIs among individuals with SCI/D living in the community range from 26 to 54%, generally within a 1-year reporting period [2]. Despite optimized prevention strategies, the majority of people with SCI/D will develop a PI during their lifetime [2].

According to the European Pressure Ulcer Advisory Panel, PIs are categorized in four stages of severity [5]. While stage I and II PI are mostly treated conservatively, surgery is generally recommended for stage III and IV because of prolonged time needed for closure and their high recurrence rate [1, 2]. Surgical management is recommended to treat stage III and IV PIs effectively and improve health and quality of life of individuals with complex PIs [1]. Still, postoperative complications are high and usually range between 30% and 50% [6–8]. Furthermore, postoperative complications such as wound dehiscence, necrosis, hematoma, bleeding, or infection increase length of hospital stay and costs [7, 9]. These complications are categorized into minor and major complications [10]. Major complications are defined as complications requiring reoperation, whereas minor complications can be treated conservatively [10]. Major complications occur in approximately 16% of the affected individuals [8, 9, 11]. The success of flap surgery depends on as well as pre- and postoperative care [1]. The treatment of stage III and IV PI in people with SCI/D must focus not only on the wound, but also on the individual’s biologic, psychologic and social system [12]. Therefore, treatment approaches require a multi-layered, coordinated involvement of different disciplines and professions such as paraplegiologists, surgeons, infectious disease specialists, physical and occupational therapists, nutritionists, and psychologist [1, 3, 6–8]. In addition to debridement and flap surgery, multidisciplinary treatment approaches generally also include other treatment elements, such as pressure relief and immobilization, infection control, wound conditioning and risk screening [13]. It is still unknown which risk factors are associated with major complications after flap surgery using a treatment approach [14, 15]. To further improve multidisciplinary treatment approaches and reduce major complications after flap surgery, we need to know what modifiable risk factors for major complications need to be considered when treating stage III and IV PI using a multidisciplinary treatment approach. Therefore, the aim of this study is to identify modifiable risk factors in people with SCI/D and stage III and IV PI associated with major complications after flap surgery.

## Methods

### Study design and study setting

We conducted an exploratory retrospective cohort study based on routinely collected clinical data in a Swiss acute and rehabilitation hospital specialized in SCI/D. At this hospital, the Basel Decubitus Approach (previously called Basel Decubitus Concept) is applied [6]. This is a multidisciplinary treatment approach developed by Lüscher et al. in the late 1990^ies^ [16]. The approach was extended according to the bio-psycho-social model of the International Classification of Functioning, Disability and Health (ICF) of the World Health Organization (WHO) [17]. The Basel Decubitus Approach includes several treatment elements: PI classification, debridement, flap surgery, pressure relief and immobilization, infection control, treatment of osteomyelitis, wound conditioning, risk screening and optimization of comorbidities, physical and occupational therapy, nutritional therapy, psychology, spasticity control, as well as prevention and education [6, 13].

### Eligibility criteria

We collected data from all consecutively admitted adults with SCI/D who were hospitalized for the first time for a stage III or IV PI over sacrum/coccyx, ischium or trochanter between January 1, 2016 and December 31, 2022. We also collected the data from subsequent treatment procedures of these individuals. If an individual had more than one stage III or IV PI over ischium, trochanter or sacrum/coccyx, each PI treatment was counted as separate treatment procedure. Data were re-collected for each treatment procedure to address changes in individuals. Exclusion criteria were pre-established. Therefore, we excluded individuals who denied the retrospective use of their data, individuals <18 years of age, individuals undergoing initial rehabilitation, and individuals with other neurological or malignant diseases. In addition, we excluded individuals who were treated conservatively or transferred to another hospital to complete treatment. Individuals who died during treatment from another condition unrelated to the PI or postoperative complication were also excluded. To determine the cause of death, we reviewed the patient’s medical record. Death that occurred more than two weeks after surgery was not considered a postoperative complication. Finally, twelve individuals with minor complications were excluded.

### Data collection

We collected sociodemographic data as well as data on participant and PI characteristics, laboratory values, comorbidities and surgical characteristics.

The neurological impairment, level of lesion, and completeness was documented with the ASIA/ISCoS International Standard for Neurological Classification of SCI (ISNCSCI) [18]. PI characteristics included PI stage according to the European Pressure Ulcer Advisory Panel [5]. Localization of PI over coccyx/sacrum, ischium and trochanter, number of PIs in this localization as well as the presence of further stage III and IV PIs in another localization such as ankle, foot or elbow were collected.

Laboratory values were from the day of hospital admission, but no later than one day after admission if no samples were taken on the day of admission. Kidney function was described by the glomerular filtration rate according to cystatin formula.

Comorbidities were recorded from the diagnosis list and hospital discharge report. Smoking status before entrance was recorded from the diagnosis list or documented anamnesis. During the study period, spasticity was not part of the Basel Decubitus Approach. Therefore, spasticity was only recorded as present or absent according to the documentation. We used the normal BMI formula (kg/m^2^), although it is not fully appropriate for individuals with SCI/D [19] because of the lack of feasible and meaningful alternative BMI adjustments [20]. If comorbidities, smoking, or spasticity were not described anywhere, we coded them as not present.

Surgical procedures were collected from discharge or surgical reports. Complications after surgery were divided into major and minor complications according to the classification of Dindo et al. depending on whether further surgical procedure was required [10]. Data were retrieved from various electronic databases used in clinical routine: KIS (Nexus, Switzerland), WicareDoc (Wigasoft, Switzerland), d.3one (D.velop, Switzerland), and ixserve.4 (ixmid software technologie GmbH, Germany).

### Statistical analysis

We performed descriptive analysis of patient, surgery and PI related factors. Categorical variables were presented as count (N) and percentage (%) and continuous data as median and interquartile range (IQR) according to the Shapiro-Wilk test. Global p-values were computed using likelihood ratio tests adjusted for clustering of PIs in individuals using random effects. A two-sided *p* value of <0.05 was considered as statistically significant. We analyzed the data using the statistical program StataSE-16 (64-bit) windows.

## Results

### Study population

**Fig. 1:**
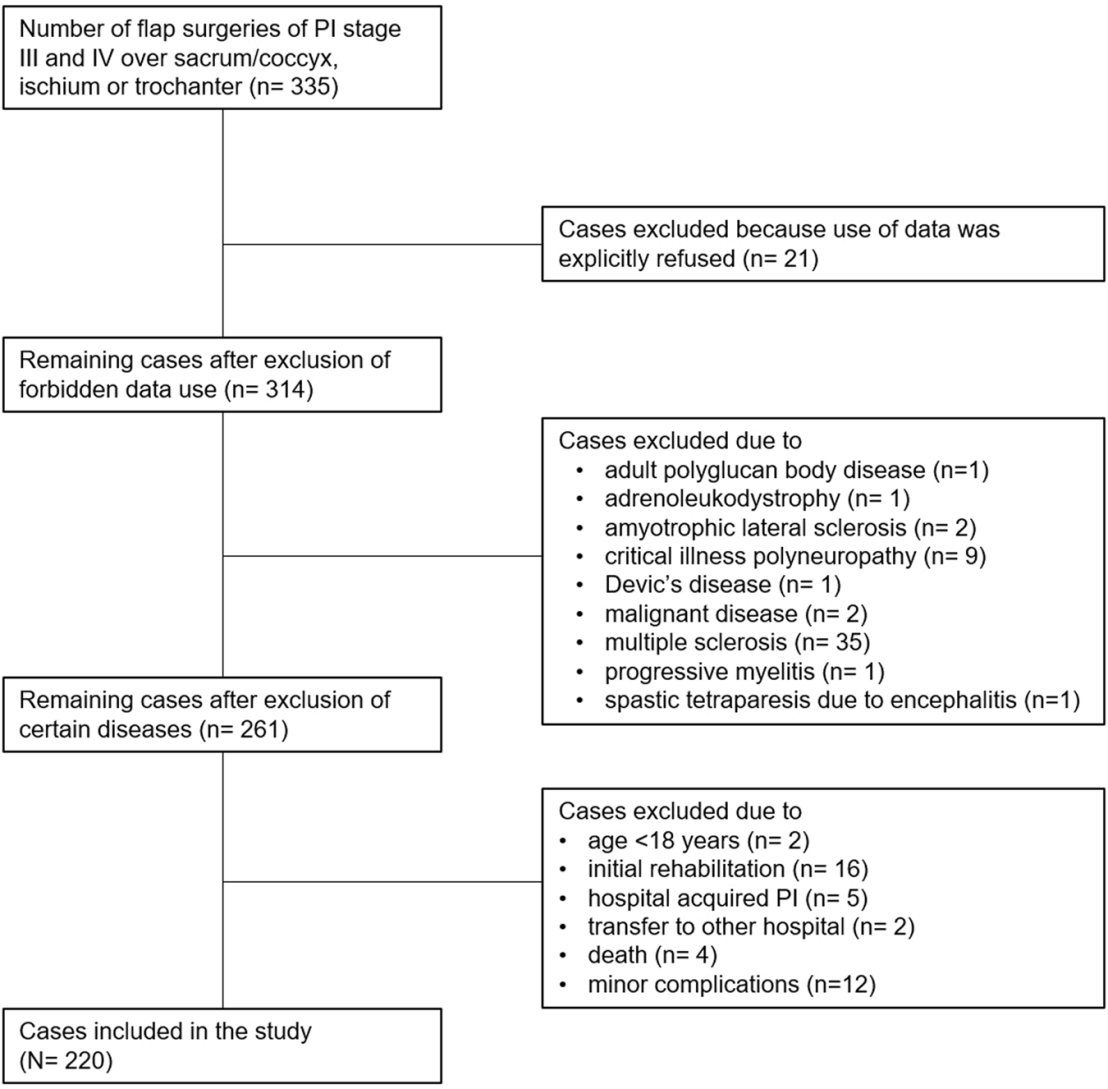
Flow chart regarding case selection. Flow chart showing the number of included cases (treatment procedures) after excluding cases with forbidden data use, individuals with certain diseases and other criteria.

Between January 1, 2016 and December 31, 2021, 335 flap surgeries for stage III and IV PI over the sacrum/coccyx, ischium or trochanter were performed on individuals with SCI/D undergoing flap surgery. We excluded 21 individuals who refused data use and 12 individuals with minor complications. Four individuals died during the immobilization phase four to six weeks postoperatively because of the following medical complications: pneumonia and cardiac failure. One individual died nine months after flap surgery due to pre-existing renal failure and pneumonia. Finally, we included 220 treatment procedures of 149 individuals (Fig. 1).

### Person and pressure injury characteristics

The study population consisted of 163 (74%) men, 133 (60%) individuals with traumatic SCI, 198 (90%) with paraplegia, 166 (75%) with an AIS A, 136 (58%) individuals with stage IV PI, 93 (42%) with osteomyelitis and 34 (15%) current smoker. Eighty-five (39%) individuals had a recurrent PI over the sacrum/coccyx, ischium or trochanter. Of those, 45 (53%) were at the same location and 40 (47%) in a different site than the previous PI. One-hundred-three individuals (69%) were treated once for PI during the observation period, 30 (20%) individuals had two recurrences, and 16 (11%) had three or more recurrences (Appendix Table 1). PIs were most commonly localized over the ischium (118 individuals, 54%) followed by coccyx/sacrum (64 individuals, 29%), and trochanter (38 individuals, 17%). Thirty-nine (18%) individuals had also a further PI at another localization (Table 1). We found 42 (19%) major complications in our study population.

**Table 1:** Patients characteristics, by complications.

Patient characteristics	Total *N* = 220 n (%)	No complication *N* = 178 n (%)	Major complication *N* = 42 n (%)	*p* value
Sex				0.50
Male	163 (74)	133 (75)	30 (71)	
Female	57 (26)	45 (25)	12 (29)	
Etiology of SCI/D				0.68
* Traumatic SCI*	*133 (60)*	*106 (60)*	*27 (64)*	
Transport activity	62 (28)	48 (27)	14 (52)	
Sports and leisure activity	30 (14)	22 (12)	8 (30)	
Fall	25 (11)	22 (12)	3 (11)	
Violence	4 (2)	4 (2)	0	
Caused by surgical intervention	10 (5)	7 (4)	3 (27)	
Other accident cause	12 (5)	10 (6)	2 (7)	
* Non-traumatic SCI*	*58 (26)*	*47 (26)*	*11 (26)*	
Inflammation/Infection	3 (1)	3 (2)	0	
Bleeding	2 (1)	2 (1)	0	
Congenital	9 (4)	9 (5)	0	
Other disease undefined	34 (15)	26 (15)	8 (73)	
* Unknown cause*	*29 (13)*	*25 (14)*	*4 (10)*	
Neurological level of SCI				0.96
C1–C4	22 (10)	18 (10)	4 (9)	
C5–C8	48 (22)	38 (21)	10 (24)	
T1–S5	150 (68)	122 (69)	28 (67)	
ASI grade				0.89
A	166 (75)	134 (75)	32 (76)	
B	32 (15)	27 (20)	5 (12)	
C	15 (8)	12 (7)	3 (7)	
D at any level	7 (3)	5 (3)	2 (5)	
PI stage				**0.01**
III	84 (42)	74 (42)	10 (24)	
IV	136 (58)	104 (58)	32 (76)	
Localization of the PI				0.13
Coccyx/Sacrum	64 (29)	46 (26)	18 (43)	
Ischium	118 (54)	102 (57)	16 (38)	
Trochanter	38 (17)	30 (17)	8 (19)	
Number of PIs at this localization				0.24
1	155 (70)	121 (68)	34 (81)	
2	51 (23)	43 (24)	8 (19)	
3	9 (4)	9 (5)	0	
4 or 5	5 (2)	5 (3)	0	
A further PI at another localization				0.25
No	181 (82)	149 (84)	32 (76)	
Yes	39 (18)	29 (16)	10 (24)	
Recurrence of previous PI				0.42
No	135 (61)	112 (63)	32 (63)	
Yes	85 (39)	66 (37)	19 (37)	
At same location	45 (53)	33 (50)	12 (63)	
At other location	40 (47)	33 (50)	7 (37)	
Osteomyelitis				**0.031**
No	127 (58)	96 (54)	31 (74)	
Yes	93 (42)	82 (46)	11 (26)	
Smoking				0.42
No	186 (85)	153 (86)	33 (79)	
Yes	34 (15)	25 (14)	9 (21)	
Age at admission (year): median (IQR)*	62 (22)	59 (22)	66 (19)	0.12
Years post injury: median (IQR)*	23 (24)	23 (24)	19 (23)	0.51
Immobilization (days): median (IQR)*	42 (16)	41.5 (14)	62 (39)	**0.0089**
Hospital stay (days): median (IQR)*	89 (53)	85 (51)	124 (68)	**0.0001**

*ISNCSCI* International standard for neurological classification of spinal cord injury, *IQR* interquartile range, *SCI/D* spinal cord injury or spinal cord disease.

*Median (IQR), since the data are not normally distributed according to the Shapiro-Wilk test.

We found a statistically significant difference between those with and without major complications in terms of PI stage (*p* < 0.01), osteomyelitis (*p* < 0.031), length of immobilization (*p* < 0.0089), and length of hospital stay (*p* < 0.0001) (Table 1).

### Comorbidity characteristics

Seventy (32%) of the individuals with stage III and IV PI had obstructive sleep apnea syndrome (OSAS), 69 (31%) had increased spasticity, and 49 (22%) had diabetes. In addition, 65 (30%) individuals had hypertonia and 63 (29%) had a urinary tract infection (UTI) during hospital stay. Of the 85 individuals with a recorded BMI, 29 (34%) were overweight or obese. Moreover, no statistically significant difference was found in comorbidities between individuals with and without major complications (Table 2).

**Table 2:** Comorbidities, by complications.

Comorbidity	Total *N* = 220 n (%)	No complications *N* = 178 n (%)	Major complications *N* = 42 n (%)	*p* value
Diabetes mellitus	49 (22)	38 (21)	11 (26)	0.53
Psychiatric diagnose	19 (11)	19 (11)	0	.
OSAS	70 (32)	58 (33)	12 (29)	0.65
Pneumonia	15 (7)	12 (7)	3 (7)	0.88
UTI	63 (29)	49 (28)	14 (33)	0.51
Vascular comorbidity	36 (16)	28 (16)	8 (19)	0.93
Hypertension	65 (30)	51 (29)	14 (33)	0.62
Osteoarthritis	25 (11)	23 (13)	2 (5)	0.13
Scoliosis	32 (15)	27 (15)	5 (12)	0.76
Amputation	26 (12)	20 (11)	6 (14)	0.76
Spasticity	69 (31)	51 (29)	18 (43)	0.37
*BMI*	*85 (39)*			1
<18.5 kg/m^2^	13 (15)	12 (17)	1 (2)	
18.5–24.9 kg/m^2^	43 (51)	38 (54)	5 (12)	
25–29.9 kg/m^2^	20 (24)	14 (20)	6 (14)	
≥30 kg/m^2^	9 (11)	7 (10)	2 (5)	

*BMI* body mass index, *OSAS* obstructive sleep apnea syndrome, *UTI* urinary tract infection.

### Blood values

We found a high percentage of pathological blood values for increased c-reactive protein (92%), erythrocyte sedimentation rate (85%), transferrin (78%), reduced HbA1c (78%), glomerular filtration rate according to cystatin formula (75%), hemoglobin (74%), serum creatinine (73%), iron (71%), 25-OH-vitamin D (68%), and cystatin C (65%) (Table 3).

**Table 3:** Blood values, by complications.

Blood values	Reference value	Total *N* = 220 n (%)	No complications *N* = 178		Major complications *N* = 42		*p* value
			not pathologic n (%)	pathologic n (%)	not pathologic n (%)	pathologic n (%)	
Albumin	32–50 g/l	132 (60)	82 (77)	24 (23)	21 (81)	5 (19)	0.92
Prealbumin	0.18–0.45 g/l	20 (9)	5 (36)	9 (64)	4 (67)	2 (33)	0.87
Total protein	64–83 g/l	122 (55)	64 (65)	34 (35)	18 (75)	6 (25)	0.42
25-OH-vitamin D	≥75 nmol/l	120 (55)	27 (28)	68 (72)	12 (48)	13 (52)	0.19
CRP	<5 mg/l	209 (95)	14 (8)	155 (91)	3 (8)	37 (93)	0.83
Sodium	135–145 mmol/l	218 (99)	129 (73)	47 (27)	30 (71)	12 (29)	0.68
Potassium	3.5–5.1 mmol/l	217 (99)	166 (95)	9 (5)	39 (93)	3 (7)	0.51
Iron	8.8–27 μmol/l	31 (14)	6 (26)	17 (74)	3 (38)	5 (63)	1
Ferritin	15–150 μg/l	108 (49)	43 (50)	43 (50)	13 (60)	9 (41)	0.56
Transferrin	2.2–3.7 g/dl	27 (12)	2 (10)	18 (90)	4 (57)	3 (43)	1
ESR	<20 mm/h	114 (52)	13 (15)	75 (85)	4 (15)	22 (85)	0.82
Creatinine	59–104 μmol/l	204 (93)	43 (26)	122 (74)	13 (33)	26 (67)	0.47
Cystatin C	0.61–0.95 mg/l	192 (87)	60 (39)	93 (61)	8 (21)	31 (79)	**0.028**
eGFR^a^	≥90 ml/min	195 (89)	43 (28)	113 (72)	6 (15)	33 (85)	0.16
Glucose	3.6 bis 5.8 mmol/l	152 (69)	53 (45)	66 (55)	20 (61)	13 (39)	0.13
HbA1c	4.8–5.9%	63 (29)	9 (17)	43 (83)	5 (45)	6 (55)	**0.033**
Hemoglobin	male: 140–170 g/l female: 120–170 g/l	219 (100)	47 (27)	130 (73)	11 (26)	31 (74)	0.92
Calcium	2.2–2.6 mmol/L	97 (44)	58 (74)	20 (26)	10 (53)	9 (47)	**0.048**
LDL cholesterol	0.2–4.0 mmol/L	34 (15)	25 (93)	2 (7)	7 (100)	0	.
HDL cholesterol	0.9–2.0 mmol/L	98 (45)	54 (68)	26 (33)	13 (72)	5 (28)	0.95
Total cholesterol	<5.2 mmol/L	102 (46)	67 (82)	15 (18)	16 (80)	4 (20)	0.91
Triglyceride	0.1–2.3 mmol/L	105 (47)	72 (85)	13 (15)	14 (70)	6 (30)	0.25
Vitamin B12	200–1000 ng/L	117 (53)	75 (81)	18 (19)	13 (54)	11(46)	**0.0049**
Folic acid	5 bis 20 μg//L	115 (52)	48 (52)	45 (48)	14 (63)	8 (36)	0.64
TSH	0.4–4.0 mU/L	113 (51)	77 (87)	12 (13)	23 (96)	1 (4)	0.16
INR	0.9–1.3	197 (90)	135 (84)	25 (16)	31 (84)	6 (16)	0.99

*CRP* C-reactive protein, *ESR* erythrocyte sedimentation rate, *eGFR* estimated glomerular filtration rate, *HbA1c* hemoglobin A1c, *HDL cholesterol* High density lipoprotein cholesterol, *INR* International normalized ratio, *LDL cholesterol* low density lipoprotein cholesterol, *TSH* thyroid-stimulating hormone.

^a^eGFR according to CKD-EPI formula.

In addition, statistically significant differences were found in cystatin c (*p* < 0.028), calcium (*p* < 0.048), HbA1c (*p* < 0.033) and vitamin B12 (*p* < 0.005) between individuals with and without major complications (Table 3). Major complications occurred more often in individuals with significantly increased cystatin c levels (31 individuals, 25%), calcium deficiency (9 individuals, 31%), normal HbA1c levels (5 individuals, 36%), and with vitamin B12 deficiency (10 individuals, 40%) (Appendix Table 2).

### Flap surgery and complication characteristics

Of the 220 surgical procedures for PIs, the following flaps were most frequently used: fasciocutaneous posterior thigh flaps (100 surgeries, 45%), fasciocutaneous gluteal rotation flaps (45 surgeries, 20%), and smaller local (Limberg) flaps (19 surgeries, 9%). Major complications occurred most frequently with the fasciocutaneous gluteal rotation flap with 13 complications out of 45 surgeries. However, we did not identify statistically significant differences in flap surgery type between the two groups (Table 4). Wound dehiscence (25 individuals, 11%) and partial flap or wound edge necrosis (11 individuals, 5%) were the most common reason for major complications (Appendix Table 3).

**Table 4:** Flap surgery techniques, by complications.

Flap surgery technique	Total *N* = 220 n (%)	No complications *N* = 178 n (%)	Major complications *N* = 42 n (%)	*p* value
Fasciocutaneous gluteal rotation flap	45 (20)	32 (18)	13 (31)	0.07
Fasciocutaneous posterior thigh flap	100 (45)	84 (47)	16 (38)	0.29
Fasciocutaneous tensor fascia lata perforator flap	4 (2)	4 (2)	0	.
Lateral advancement flap	16 (7)	14 (8)	2 (5)	0.44
Local (Limberg) flap	19 (9)	16 (9)	3 (7)	0.64
Other	34 (15)	26 (15)	8 (19)	0.42
Unknown	2 (1)	2 (1)	0	.

## Discussion

We examined 60 risk factors for postoperative complications in a setting where the Basel Decubitus Approach for individuals with SCI/D and PIs is established. We detected a high number of internal and paraplegiological comorbidities such as sleep apnea, diabetes or hypertonia in the study population. Major complications occurred more often in individuals with stage IV PI, individuals without osteomyelitis, and individuals with pathological blood concentrations of cystatin c, calcium, and vitamin B12 as well as normal blood concentrations of HbA1c.

The discussion will focus on the need of a multidisciplinary treatment approach and mainly on risk factors that were statistically significant in the Basel Decubitus Approach. The incidence of diabetes and sleep apnea disorders was up to 30% in our cohort. In contrary to other studies, those two comorbidities were not associated with postoperative complications [8, 9, 21]. This confirms the need to treat PI in individuals with SCI/D in a setting where comorbidities can be addressed, diagnosed, and treated [6, 8].

With regard to patients’ characteristics, stage IV PI was associated with major complications. stage IV PI is more severe than stage III because the tissue damage extends into muscle, bone, tendon, or joint capsule [5]. This finding could be used to stimulate prevention strategies to encourage individuals with SCI/D to agree to early hospital admission. In addition, resources for inpatient treatment could be created to allow early admission in case of stage III PI in the sitting region.

The occurrence of osteomyelitis as a risk factor for post-surgical complication showed contradictory results. In the study by Rigazzi et al., individuals without osteomyelitis experienced significantly more post-operative complications than those with osteomyelitis [22] whereas Kreutzträger et al. observed the opposite [9]. In our study, we also found more major complications if no osteomyelitis was diagnosed. One explanation might come from the different approach to antibiotic therapy in people with osteomyelitis in the Basel Decubitus Approach [6, 13, 22]. Individuals without osteomyelitis are treated for one week with antibiotics [6, 13, 22]. In contrary, those with osteomyelitis are treated for six to eight weeks based on an individualized antibiotic selection according to resistance analyses of bone biopsies [6, 13, 22]. If bone is exposed, the surgeon takes bone samples at the time of debridement and at the time of flap reconstruction [6, 13, 22]. The bone samples are microbiologically and histopathologically examined to find antibiotic therapy appropriate for resistance [6, 13, 22]. This is never the case in stage III PIs [6, 13, 22]. In contrast, standardized antibiotics, most often amoxicillin clavulanic acid, are administered when bone is not exposed and thus osteomyelitis is not suspected [6, 13, 22]. Therefore, we hypothesize that individualized antibiotic treatment might be the reason why the presence of osteomyelitis was a protective factor for major complications. To address this problem, on the one hand, a broader antibiotic therapy could be chosen, such as the approach of the Montecatone Rehabilitation Institute [8]. On the other hand, soft tissue samples could be taken from all PIs and treated according to resistance. Complementing the analysis of tissue samples, antibiotic selection could include urinary bacteria and their resistance. Around sixty percent of all people with SCI/D suffer from chronic partly asymptomatic urinary tract infections, which should very well be treated after flap surgery [23].

In the Basel Decubitus Approach, a set of blood values are routinely assessed to determine the inflammatory situation, nutrition, renal function, and anemia. If deficits in blood values were present at admission, they were treated before flap surgery [13]. However, our study showed that blood values in particular are often not determined at the time of entry. The values might not be examined because of infection with elevated CRP values. Eighty percent of our patients had elevated inflammation values. The interpretation of pathological blood values as risk factors for post-surgical complications proved to be extremely difficult. For a correct interpretation of blood values, on the one hand, other blood values need to be considered. On the other hand, blood values can be dependent on other blood values. Individuals with major complications were significantly more likely to have pathological values of vitamin B12 (*p* < 0.005) and calcium (*p* < 0.048) at admission. Both values are dependent from kidney function and infect situation and should be interpreted and treated in the comprehensive patient situation [24, 25]. Our results indicate that renal function should be investigated and monitored more closely in the future. Renal dysfunction impairs wound healing and increases the risk of developing postoperative wound infections [26]. Decreased renal function can also led to lower vitamin B12 levels [24]. According to Rieger et al., vitamin B12 deficiency should be treated because of its involvement in the metabolically intense wound healing process [27]. It is possible that treatment with vitamin B12 was not sufficient to normalize levels by the time of flap surgery and was therefore associated with major complications. The same could apply for calcium levels. In animals, calcium deficiency delays wound healing and increases the prevalence of chronic wound formation [28, 29]. Calcium affects the wound healing process in humans as well [28]. However, ideal calcium concentrations for the different phases of wound healing are unknown [28]. In our opinion, it is important to treat calcium deficiency because individuals with normal levels had fewer complications. Although, 49 (22%) individuals had diabetes, we found no elevated HbA1c levels. Among the 63 (29%) subjects with a measured HbA1c value, those with low HbA1c levels experienced fewer major complications than those with normal HbA1c levels. One potential explanation might be that PI lead to an increased metabolism [30] which might decrease HbA1c levels like fasting or exercising [31]. Therefore, a normally adjusted HbA1c level might be falsely low. A high HbA1c value, on the other hand, might be at a normal level at the time of hospital admission due to PI. The longer a person suffers from a deep PI, the more the HbA1c level might decrease due to the increased metabolism [30]. With increased metabolism, there is also a risk of malnutrition (e.g. low body weight and poor oral food intake) [5, 32]. Therefore, in our opinion HbA1c levels should be interpreted under consideration of the length of the deep PI and the nutrition status of the patient [5, 32]. Furthermore, we assume that HbA1c levels during stage III and IV PI might not say anything about a well-controlled diabetes.

Although, BMI assessment is part of the Basel Decubitus Approach, BMI was measured infrequently and in contrast to other studies, was not statistically significant [13, 19]. The use of BMI formula is not fully appropriate for individuals with SCI/D because it does not describe body constitution and composition [19]. Therefore, it needs to be discussed which formula or measurement tool (e.g. dual-energy X-ray absorptiometry (DEXA) or skinfold thickness) should be used in the clinical setting to describe body constitution and composition to observe postoperative complication [33].

### Clinical implication

The results showed that individuals with SCI/D and stage III and IV PI often have several comorbidities as well as complex changes of metabolism and infection. In this respect, the treatment of these patients requires interdisciplinary management, so that a high level of paraplegiology, internal medicine and plastic surgery expertise are required to address the complex needs of patients with SCI/D and stage III and IV PI.

Neither diabetes nor other treatable comorbidities were significantly associated with major complications if the treatment of these factors is included in a multidisciplinary approach [8, 13]. The results might be different since the deficits or comorbidities remained untreated or insufficiently treated at the time of flap surgery.

### Limitations

We used a retrospective observational cohort design. The data were extracted from regular clinical documentation. Therefore, the quality of data on some variables was limited because some standardized documentation was lacking (e.g., spasticity, BMI, prealbumin, iron, transferrin, LDL cholesterol). Likewise, HbA1c values were only measured in 63 (29%) of the individuals. As this study was exploratory, there was no predefined hypothesis about an effect [34]. For this reason, statistical power was not a primary concern [34]. Furthermore, there may be other risk factors that could not be assessed retrospectively, such as periodic limb movements during sleep (PLMS), vascular status, autonomic nervous system function, and immunosuppression. Another limitation of this study is the examination of risk factors in the context of a special setting using a treatment approach. Thus, the results are not generalizable to a setting without a similar treatment approach. Moreover, there is a limitation in the alpha error accumulation due to multiple testing. A further limitation is the interpretation of the blood values because the individual blood values as such cannot be interpreted in the overall context. For future research, a model for blood values would be needed to observe the association of blood values on complication. Furthermore, a risk prediction model should be developed to observe the association of various risk factors on major complications. We also recommend to collect data prospectively.

## Conclusion

In the Basel Decubitus Approach, the absence of osteomyelitis, pathological levels of cystatin c levels, calcium and vitamin B12 levels as well as normal HbA1c levels were associated with major complications after flap surgery. Thus, in cases of pathological levels of cystatin c, calcium and vitamin B12 levels it might be useful to delay flap reconstruction until the values are improved. The role of normal HbA1c levels in relation to major complications needs further discussions. Individualized antibiotic treatment based on tested tissue samples in individuals with and without osteomyelitis might help decreasing complication rates. For future analysis of the impact of several risk factors on major complications we recommend a risk prediction model.

### Reporting summary

Further information on research design is available in the Nature Research Reporting Summary linked to this article.

### Supplementary information

Supplementary files

Reporting Summary

## Data Availability

All data are stored with the corresponding author and can be asked directly (carina.faehndrich@paraplegie.ch).
