# Supplementary material for: Risk factors of major complications after flap surgery in the treatment of stage III and IV pressure injury in people with spinal cord injury/disorder: a retrospective cohort study
Source: Spinal Cord. 2023 Dec 20;62(1):34–41. doi: 10.1038/s41393-023-00944-9 (PMC10783547; doi:10.1038/s41393-023-00944-9)
Supplement: Supplementary file 1 — Supplementary files [file 41393_2023_944_MOESM1_ESM.pdf]

Appendix Table 1. Number of PI during the observation period

Number of PI during observation period	Number of individuals N=149 n (%)	Total cases N=220 n (%)
1	103 (69)	103 (47)
2	30 (20)	60 (27)
3	12 (8)	36 (16)
4	2 (1)	8 (4)
5	1 (1)	5 (2)
6	0	0
7	0	0
8	1 (1)	8 (4)

Appendix Table 2. Statistically significant blood values categorized in levels, by complications

Blood values (reference value)	Total N=220 n (%)	Major complications	
		No N=178 n (%)	Yes N=42 n (%)
Cystatin C (0,61-0,95 mg/l)			
Deficit	0	0	0
Norm	68 (31)	60 (88)	8 (12)
Excess	124 (56)	93 (75)	31 (25)
Calcium (2.2-2.6 mmol/L)			
Deficit	29 (13)	20 (69)	9 (31)
Norm	68 (31)	58 (85)	10 (15)
Excess	0	0	0
HbA1c (4.8-5.9%)			
Deficit	37 (58)	34 (92)	3 (8)
Norm	14 (22)	9 (64)	5 (36)
Excess	12 (19)	9 (75)	3 (25)
Vitamin B12 (200-1000 ng/L)			
Deficit	25 (11)	15 (60)	10 (40)
Norm	88 (40)	75 (85)	13 (15)
Excess	4 (2)	3 (75)	1 (25)

Appendix Table 3. Characteristics of major complications

Type of complication N=220	Major complications N=42 n (%)
Wound dehiscence	25 (11)
Wound infection	1 (0)
Bleeding or hematoma	4 (2)
Necrosis	11 (5)
Unknown	1 (0)
